# The Dipeptidyl Peptidase-4 Inhibitor Sitagliptin Protects against Dyslipidemia-Related Kidney Injury in *Apolipoprotein E* Knockout Mice

**DOI:** 10.3390/ijms150711416

**Published:** 2014-06-26

**Authors:** Jingjing Li, Meiping Guan, Chenzhong Li, Fuping Lyv, Yanmei Zeng, Zongji Zheng, Chengzhi Wang, Yaoming Xue

**Affiliations:** Department of Endocrinology and Metabolism, Nanfang Hospital, Southern Medical University, Guangzhou 510150, China; E-Mails: lijj6116@163.com (J.L.); mpguan@163.com (M.G.); xichongli@163.com (C.L.); lv.fu.ping@163.com (F.L.); emilyzengym@163.com (Y.Z.); achloe@sina.cn (Z.Z.); weiwei7408@hotmail.com (C.W.)

**Keywords:** sitagliptin, HFD, dyslipidemia, kidney injury, AMPK, Akt, TGF-β1, FN, p38/ERK MAPK

## Abstract

The goal of this study was to investigate the possible protective effects of sitagliptin against dyslipidemia-related kidney injury in *apolipoprotein E* knockout (apoE^−/−^) mice. Eight-week-old male apoE^−/−^ mice were randomized to receive either a high fat diet (HFD, apoE^−/−^ group) or HFD mixed with sitagliptin (sita + apoE^−/−^ group) for 16 weeks. A control group of age- and gender-matched C57BL/6J mice were fed a HFD. The apoE^−/−^ group exhibited increases in body weight and serum lipid levels in addition to high-density lipoprotein, and increases in 24-h urinary 8-hydroxy-2-deoxyguanosine and albuminuria excretion. Decreased insulin sensitivity was also observed in the apoE^−/−^ group. These mice additionally contained enlargements of the glomerular mesangial matrix area, lipid deposition area, and renal interstitium collagen area. The apoE^−/−^ group also demonstrated down-regulation of phosphorylated AMP-activated protein kinase (AMPK), increases in renal mRNA expression of *transforming growth factor-beta 1* (TGF-β1) and *fibronectin* (FN), and increased protein expression of Akt, TGF-β1, FN and p38/ERK mitogen-activated protein kinase (MAPK). Sitagliptin treatment successfully ameliorated all the deleterious effects of dyslipidemia tested. To our knowledge, this is the first time that sitagliptin has been shown to reverse the renal dysfunction and structural damage induced by dyslipidemia in apoE^−/−^ mice. Our results suggest that the renoprotective mechanism of sitagliptin may be due to a reduction in Akt levels, a restoration of AMPK activity, and inhibition of TGF-β1, FN, and p38/ERK MAPK signaling pathways.

## 1. Introduction

Dyslipidemia is one of the major risk factors for the progression of chronic kidney disease. This abnormal lipid metabolism is mainly manifested as elevated serum cholesterol levels, elevated triglyceride levels and altered apolipoprotein profile [1,2,3]. Recent investigations have suggested a correlation between the pathogenesis of primary kidney diseases and dyslipidemia. Dyslipidemia has also been reported to cause the worsening of renal function in patients with pre-existing nephropathies [4,5,6]. Additionally, in nondiabetic patients presenting with proteinuria, the presence of elevated serum cholesterol and triglycerides levels resulted in a nearly two-fold increase in the rate of kidney failure, as compared to normolipidemic patients [7].

*Apolipoprotein E* knockout (apoE^−/−^) mice are a well-accepted animal model of hyperlipidemia, and have been used extensively to study the effects of this disease on atherosclerosis and renal injury [8]. In apoE^−/−^ mice, dyslipidemia-related kidney injury is associated with remarkable pathological alterations, including lipid deposition at the glomerulus, an expanded meangium, and an accumulated extracellular matrix (ECM).

Previous studies have demonstrated that overexpression of transforming growth factor-beta 1 (TGF-β1) results in increased fibronectin (FN) synthesis and an activated mitogen-activated protein kinase (MAPK) signaling pathway. In patients with diabetic or many other renal injuries, up-regulation of TGF-β1, FN, and the MAPK signaling pathway are jointly associated with renal fibrosis and glomerular sclerosis. Furthermore, AMP-activated protein kinase (AMPK) is an enzyme ubiquitously expressed in the kidney and other organs [9]. It is recognized as the key molecule of energy regulation in response to metabolic stresses, such as glycometabolic and lipometabolic disorders. A growing body of evidence has demonstrated that decreased AMPK phosphorylation induced by dyslipidemia plays an important role in the progression of kidney damage [9,10,11,12]. AMPK phosphorylation is also decreased in kidney under conditions of high glucose and this phosphorylation is partly restored by inhibition of Akt, suggesting that AMPK is downstream of Akt [13]. Inhibition of AMPK by Akt has also been observed in cardiac myocytes [14].

Sitagliptin, a dipeptidyl peptidase-4 (DPP-4) inhibitor, is a new class of antidiabetic agents. DPP-4 is a peptidase responsible for degradation of incretin hormones like glucagon-like peptide-1 (GLP-1) and glucose-dependent insulinotropic polypeptide (GIP). Accordingly, sitagliptin treatment exerts its biological effects by increasing the physiological concentration of GLP-1 [15]. GLP-1 binds to GLP-1 receptor, a G-protein-coupled receptor that is expressed in numerous tissues including the glomeruli and renal tubule [16]. The receptor of GIP is not expressed in the kidney [17]. The sitagliptin-induced increase in GLP-1 results in insulin secretion in a glucose-dependent manner, and increased GLP-1 promotes pancreatic β cell proliferation [18]. Concurrently, sitagliptin impacts the synthesis and secretion of blood lipids [19,20,21,22]. Recent studies have demonstrated sitagliptin treatment is able to attenuate renal damage and reduce urinary albumin excretion in diabetic animal models [23,24,25]. In addition to its protective effects in diabetic nephropathy (DN), sitagliptin also protects the kidney against ischemia reperfusion injury and acute kidney injury [26,27].

Due to these protective effects of sitagliptin, we hypothesized that sitagliptin could alleviate dyslipidemia-related renal injury in apoE^−/−^ mice. We additionally hypothesized that this protective effect may be associated with the phosphorylation of AMPK, inhibition of Akt, TGF-β1 and FN expression, and inhibition of the MAPK signaling pathway.

## 2. Results

### 2.1. Effect of Sitagliptin on Body Weight, Blood Glucose Level, Serum Lipid Level, and Insulin Sensitivity

**Figure 1:**
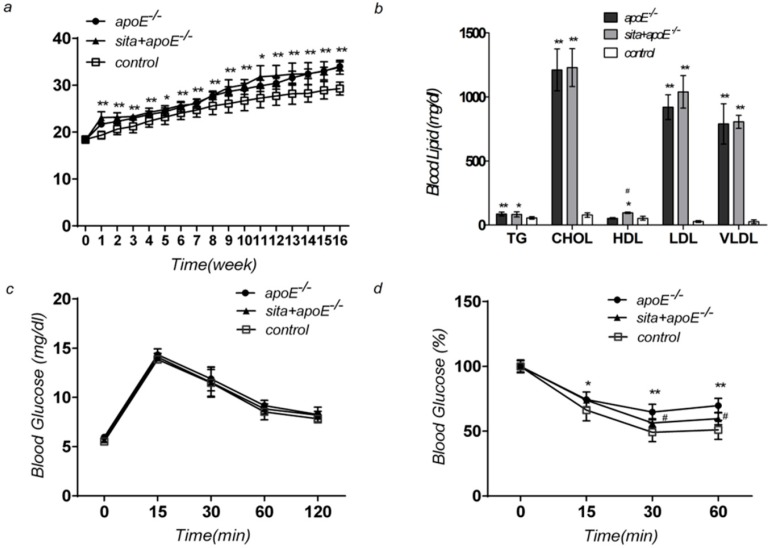
Effect of sitagliptin on body weight, blood glucose level, serum lipid level, and insulin sensitivity. ApoE^−/−^, sita + apoE^−/−^ and control groups were tested at week 16 for changes in (**a**) body weight; (**b**) serum lipid; (**c**) intraperitoneal glucose tolerance test; (**d**) intraperitoneal insulin tolerance test. Values are reported as mean ± S.D. * *p* < 0.05 and ** *p* < 0.01 *vs.* control group at same time points; ^#^ *p* < 0.05 *vs.* sita + apoE^−/−^ group.

Starting at week one, significant increases in body weight were observed in the apoE^−/−^ and sita + apoE^−/−^ group, as compared to the control group (Figure 1a) (*p* < 0.05). Both the sita + apoE^−/−^ group and apoE^−/−^ group demonstrated markedly elevated levels of triglyceride (TG), cholesterol (CHOL), low-density lipoprotein (LDL), and very low-density lipoprotein (VLDL), as compared to the control group (Figure 1b) (*p* < 0.01). A slight, but significant increase in high-density lipoprotein (HDL) levels was seen in sita + apoE^−/−^ group. No significant differences were seen in fasting glucose levels between the three groups (Figure 1c) (*p* < 0.05). Sitagliptin treatment significantly increased insulin sensitivity as compared to apoE^−/−^ group (Figure 1d) (*p* < 0.05).

### 2.2. Effect of Sitagliptin on 24-h Urinary Albumin and 8-Hydroxy-2-deoxyguanosine (8-OHdG) Excretion

**Figure 2:**
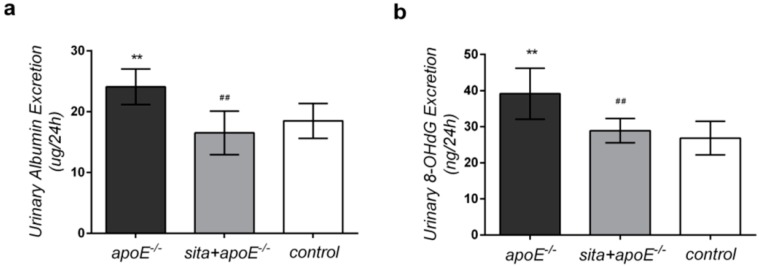
Effect of sitagliptin on 24-h urinary albumin and 8-hydroxy-2-deoxyguanosine (8-OHdG) excretion. ApoE^−/−^, sita + apoE^−/−^ and control groups were tested at week 16 for (**a**) 24-h urinary albumin excretion; (**b**) 24-h urinary 8-OhdG excretion. Values are mean± S.D. ** *p* < 0.01 *vs.* control group; ^##^ *p* < 0.01 *vs.* apoE^−/−^ group.

The effects of dyslipidemia in the context of sitagliptin treatment were next examined by quantifying urinary albumin and 8-hydroxy-2-deoxyguanosine (8-OHdG) excretion levels (Figure 2). A 24-h urine collection assay demonstrated that sitagliptin treatment resulted in a 1.458-fold reduction in albumin levels in apoE^−/−^ mice (24.074 ± 2.940 µg in apoE^−/−^ *vs.* 16.510 ± 3.583 µg in sita + apoE^−/−^) (Figure 2a). Sitagliptin treatment also resulted in a 1.355-fold decreased in 8-OH-dG excretion in apoE^−/−^ mice (39.127 ± 7.064 ng in apoE^−/−^ *vs.* 28.883 ± 3.359 ng in sita + apoE^−/−^) (Figure 2b). There were no significant differences in urinary albumin and 8-OH-dG levels between the sita + apoE^−/−^ group and control group (*p* < 0.05).

### 2.3. Effect of Sitagliptin on Dyslipidemia-Induced Pathological Changes of the Kidney

**Figure 3:**
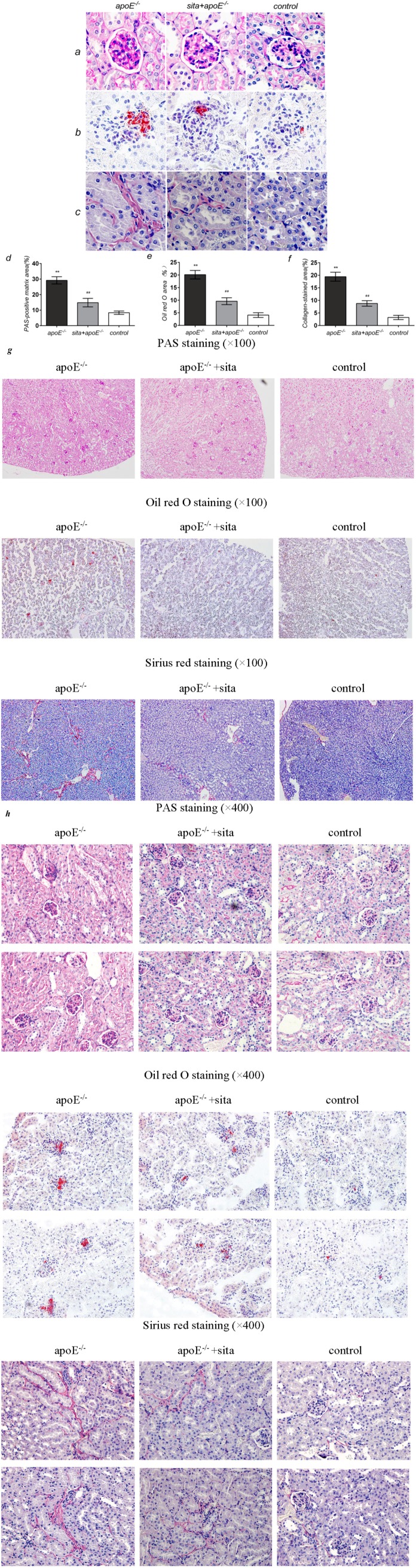
Effect of sitagliptin on dyslipidemia-induced pathological changes of the kidney. Histochemical changes in kidney tissue were observed in the apoE^−/−^, sita + apoE^−/−^ and control groups. (**a**) PAS staining for glomerular mesangial matrix; (**b**) Oil-red O staining for lipid in the renal cortical glomeruli; (**c**) Sirius red staining for collagen in the renal interstitium. Representative photomicrographs are shown at 400× magnification; Quantitative assessment of (**d**) mesangial matrix accumulation; (**e**) lipid deposition; (**f**) collagen proliferation. Values are mean ± S.D. ** *p* < 0.01 *vs.* control group; ^##^ *p* < 0.01 *vs.* apoE^−/−^ group; (**g**) Full representative photomicrographs of PAS staining, Oil-red O staining and Sirius red staining are shown at 100× magnification; (**h**) Full representative photomicrographs of PAS staining, Oil-red O staining and Sirius red staining are shown at 400× magnification.

Kidney samples were collected at the end of the experiment and examined for renal pathological changes. As compared to the apoE^−/−^ group, sitagliptin treatment significantly reduced glomerular mesangial matrix accumulation, as evidenced by decreased Periodic Acid-Schiff (PAS)-positive areas (Figure 3a,d) (*p* < 0.01). Oil-red O staining showed remarkably increased glomerulus lipid deposition in the apoE^−/−^ group and this effect was significantly reduced by sitagliptin treatment (Figure 3b,e) (*p* < 0.01). Similarly, collagen proliferation in the renal interstitium, as determined by Sirus red staining, was distinctly increased in the apoE^−/−^ group (Figure 3c,f). Sitagliptin treatment significantly reduced this proliferation in the sita + apoE^−/−^ group (*p* < 0.01). No significant differences were seen between sita + apoE^−/−^ and control groups for any of the parameters tested.

### 2.4. Effect of Sitagliptin Treatment on Renal Cortical mRNA Expression of DPP-4, GLP-1R, TGF-β1, and FN

**Figure 4:**
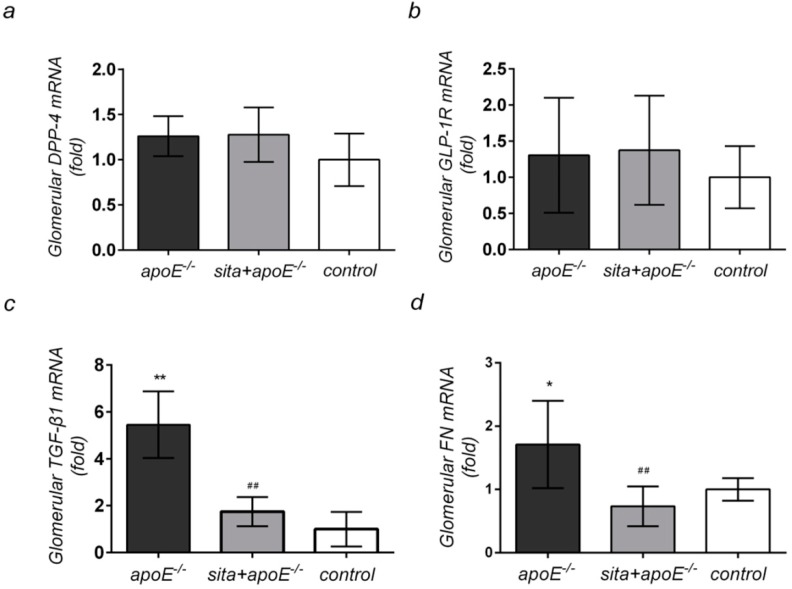
Effect of sitagliptin renal cortical mRNA expression of *DPP-4*, *GLP-1R*, *TGF-β1* and *FN* Real time RT-PCR analysis was conducted on apoE^−/−^, sita + apoE^−/−^ and control group at week 16 to examine (**a**) *DPP-4*; (**b**) *GLP-1R*; (**c**) *TGF-β1* and (**d**) *FN* expression in renal glomeruli. Values are mean ± SD * *p* < 0.05 and ** *p* < 0.01 *vs.* control group; ^##^ *p* < 0.01 *vs.* apoE^−/−^ group.

The effects of dyslipidemia on *TGF-β1* and *FN* mRNA expression in the renal cortex were examined next. Dyslipidemia resulted in increased renal cortical mRNA levels of *TGF-β1* and *FN*, as compared to the control group (Figure 4c,d). Of all pro-fibrotic markers tested, the most significant increased in mRNA expression was seen for *TGF-β1*. Oral administration of sitagliptin significantly reduced the mRNA expression of *TGF-β1* and *FN* (*p* < 0.05 and *p* < 0.01, respectively). However, no significant differences in renal cortical mRNA expression of *DPP-4* and *GLP-1R* were observed between the three groups (Figure 4a,b).

### 2.5. Effect of Sitagliptin on Renal Protein Expression of AMPK, Akt, TGF-β1, FN and MAPK Signaling Pathway

**Figure 5:**
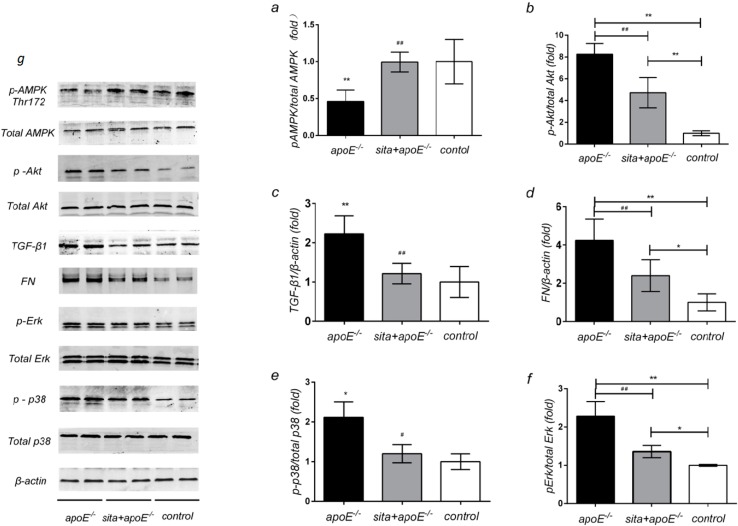
Effect of sitagliptin on renal protein expression of AMPK, Akt, TGF-β1, FN and MAPK signaling pathway. Protein lysates (30 μg) from renal cortex were separated by SDS-PAGE and analysed by Western blotting. ApoE^−/−^, sita+ apoE^−/−^ and control mice wereanalyzed for(**a**) phospho-AMPK/total AMPK; (**b**) phospho-Akt/total Akt; (**c**) TGF-β1/β-actin; (**d**) FN/β-actin; (**e**) phospho-p38/total p38; (**f**) phospho-ERK/total ERK levels. Values are means ± S.D. * *p* < 0.05 and ** *p* < 0.01 *vs.* control group, ^#^ *p* < 0.05 and ^##^ *p* < 0.01 *vs.* apoE^−/−^ group; (**g**) All the proteins were shown.

Western blot analysis showed that dyslipidemia resulted in a marked reduction in phospho-AMPK expression, as compared to control group (Figure 5a). Conversely, renal expression of phospho-Akt, TGF-βl, FN, phospho-p38, and phospho-ERK were all significantly higher in the apoE^−/−^ group, as compared to the control group. Sitagliptin treatment contributed to significant increases in phospho-AMPK expression, as well as an increase in the ratio of phospho-AMPK/total AMPK (Figure 5a) (*p* < 0.01). In contrast to its effects on AMPK, phospho-Akt, TGF-β1, and FN levels were significantly decreased following sitagliptin treatment (Figure 5b,c,d) (*p* < 0.01). Consistent with this finding, sitagliptin significantly reduced the expression of phospho-p38 (Figure 5e) and phospho-ERK (Figure 5f) (*p* < 0.05, *p* < 0.01, respectively).

## 3. Discussion

Since the hypothesis of “lipid nephrotoxicity” was raised in 1982 [28], experimental and clinical studies have suggested that dyslipidemia, specifically hypercholesterolemia, is a major independent risk factor of the kidney disease [29,30]. Lipid deposition, glomerular mesangial expansion, ECM accumulation, macrophage infiltration, and collagen proliferation are recognized as early events in the development of dyslipidemia-induced glomerulosclerosis and interstitial fibrosis [31]. These pathological changes likely begin long before the appearance of hypertension or diabetes in patients with dyslipidemia.

ApoE^−/−^ mice are a well-characterized genetic background that have been used extensively to study the effects of hyperlipidemia on renal injury, as well as cardiovascular disease and hepatic fibrosis [32]. We have previously developed the apoE^−/−^ mouse model in order to study the dyslipidemia-related renal injury and atherosclerosis [33]. In addition to affecting lipid levels, knockout of the apoE gene directly affects renal function. However, the effectiveness of using apoE^−/−^ mice to study renal disease is blunted by the fact that feeding these mice standard chow results in aortal and renal pathological features only late in life, at 24 and 36 weeks of life, respectively [8,34]. Previous studies have demonstrated that apoE^−/−^ mice fed a HFD develop aortal and renal diseases much more rapidly than if they were fed standard chow. Accordingly, the apoE^−/−^ mice were fed a HFD and developed an earlier onset of renal disease in our study. It should be noted however, that many studies have demonstrated apoE^−/−^ mice given a HFD for 16 weeks exhibit no impaired fasting glycaemia [33,35].

DPP-4 is a ubiquitous, Type II cell surface glycoprotein, and is widely expressed in all tissues. The role of DPP-4 in kidney has been investigated using the DPP-4 or GLP-1 receptor knock-out mice, in addition to DPP-4 inhibitors [36,37,38]. Studies have demonstrated that GLP-1 directly decreases blood glucose, reduces the glycation end products level, inhibits the oxidative stress reaction, and down-regulates monocyte chemoattractant protein-1 (MCP-1) and TGF-β1 levels in mesangial cells [39]. Treatment with DPP-4 inhibitors, which increase GLP-1 levels, has been shown to exert numerous renoprotective effects. These effects include a reduction in blood glucose and lipid levels, inhibition of inflammation and oxidative stress, amelioration of mesangial expansion, and an elevation of the glomerular filtration rate (GFR), among other effects. However, the effects of DPP-4 peptidase are not specific to GLP-1 and DPP-4 cleaves other proteins. In the kidneys of DPP-4^+/+^ animals, the levels of high mobility group protein-B1(HMGB1) and Meprin β, which act as pro-inflammation and ischaemia/reperfusion injury factors, were elevated and are likely substrates of DPP-4 [40,41]. In contrast, DPP-4 has been shown to degrade stromal derived growth factor-β (SDF-β) and brain natriuretic peptide (BNP) in kidneys, thus blocking the protective effects of these proteins. Therefore, DPP-4 inhibitors exert both GLP-1-dependent and independent renoprotective effects [42].

Previous studies have suggested that therapeutic intervention with a DPP-4 inhibitor is effective in postponing the development of neuropathy, cardiovascular disease, and diabetic nephropathy [25,35,43,44]. We therefore sought to determine whether sitagliptin treatment would also ameliorate dyslipidemia-related renal injury. We were additionally interested in determining the molecular mechanism by which sitagliptin treatment reduced dyslipidemia-related renal injury.

To our knowledge, our study is the first to report the ability of sitagliptin to alleviate renal dysfunction and structural damage resulting from dyslipidemia. Our results demonstrate that apoE^−/−^ mice given a HFD for 16 weeks present obvious hyperlipidemia and increased body weight, as compared to the control group. In addition, insulin sensitivity was also decreased in the apoE^−/−^ group and this effect could be mitigated by sitagliptin treatment. Interestingly, no significant differences in body weight, serum lipid (besides HDL) and fasting glucose levels were observed between aopE^−/−^ mice treated with and without sitagliptin. In the apoE^−/−^ group, 24-h urinary albumin and 8-OHdG excretion were elevated and may be the result of both glomerular and proximal tubular dysfunction, as well as renal oxidative stress injury. Treatment with sitagliptin was also able to mitigate the effects of renal injury, as evinced by decrease in urinary 8-OHdG and albumin excretion.

Consistent with previous reports [8,45], we showed that histopathological evaluation of kidney cortex tissue from apoE^−/−^ mice demonstrates obvious structural damage. These pathological features include glomerular mesangial matrix accumulation, lipid deposition and interstitum collagen proliferation. Our results suggest that sitagliptin treatment is able to reduce the mild glomerular lesions and renal inerestitum fibrosis observed in this study. This is the first time that the beneficial effects of sitagliptin treatment on dyslipidemia-related renal injury, independent of decreases in blood glucose and lipid, have been observed.

We additionally explored the expression of AMPK, Akt, TGF-β1, FN, and p38/ERK MAPK signaling pathway in the context of renal injury and sitagliptin treatment. Our results suggest that these proteins may play an important role in the protective mechanism by which sitagliptin reduces dyslipidemia-related renal injury. Previous studies have demonstrated that in rat mesangial cells, activated AMPK is able to inhibit the high glucose-induced overexpression of TGF-β1, FN, and collagen. Our results also demonstrate that sitagliptin treatment significantly increased phospho-AMPK, while reducing renal levels of TGF-β1, FN, phospho-p38 and phospho-ERK, as compared to the apoE^−/−^ group.

Many previous reports have confirmed a link between TGF-β1 expression and renal fibrosis [46,47,48,49]. These studies demonstrated that increased glomerular TGF-βl expression promotes mesangial cell proliferation, stimulates synthesis of ECM components (such as FN), and increases accumulation of the glomerular mesangial matrix [49,50,51]. These changes ultimately lead to chronic glomerulosclerosis and interstitial fibrosis [47,52,53,54]. Sitagliptin treatment has been shown to exert a renoprotective role by down-regulating TGF-β1 expression in DN. Our results presented here are supportive of a role for sitagliptin in down-regulating TGF-β1 expression. We additionally showed that expression of FN, an important component of ECM and capillary basement membranes, was upregulated in the apoE^−/−^ group. Our results demonstrate that sitagliptin treatment significantly reduced the mRNA and protein expressions of FN. The p38/ERK MAPK is the primary intracellular signal transduction pathway involved in many pathological and physiological processes. Importantly, p38/ERK MAPK also plays an important role in mesangial cell proliferation, glomerular mesangial matrix accumulation, and collagen overexpression. Studies have demonstrated that p38/ERK MAPK, a downstream effector of the TGF-β/Smad signaling pathway, is activated by TGF-β1 [55,56,57]. Therefore, it is likely that sitagliptin-induced reduction of TGF-β1 will also lead to an inhibition of FN expression and the p38/ERK MAPK signaling pathway.

Alteration of Akt activity in diabetic mellitus (DM) is associated with the pathophysiology of diabetic microvascular complications [58]. HFD-induced PPARβ expression in the kidney has also been shown to activate the phosphatidylinositol 3-kinase (PI3K)/Akt pathway, leading to oxidative stress and lipoapoptosis [59]. In the present study, we observed that apoE^−/−^ mice concurrently contain increased phospho-Akt levels and decreased phospho-AMPK levels. These results are consistent with a previously published report, which suggests that Akt regulates AMPK activity [13,14]. Our study demonstrates that sitagliptin distinctly reduced the expression of Akt.

AMPK, a conservative heterotrimeric kinase, is stimulated by an increased AMP/ATP ratio and inhibited by decreases in ATP concentrations. Considering its well-documented contribution to increased glucose uptake and fatty acid oxidation, decreased abnormal lipid deposition, and maintenance of cellular homeostasis, AMPK is recognized as a new target for the treatment of type 2 diabetes mellitus and obesity. There is a central role for AMPK in HFD-induced kidney disease, as ECM accumulation, inflammation, and lipid deposition were all mitigated by AMPK activation [60]. In addition, GLP-1 has been demonstrated to activate AMPK and subsequently contribute to pancreatic beta cell proliferation and hepatic lipogenesis suppression. Since sitagliptin has been shown to increase GLP-1 levels, we hypothesized a role for AMPK in dyslipidemia. This study demonstrates that chronic lipid deposition, as well as associated renal function and structural alterations are probably mediated by AMPK. It is worth mentioning that our results revealed that sitagliptin treatment resulted in a significant reduction in Akt phosphorylation despite enhanced phosphorylation of AMPK. These results provide new insight into the possible mechanisms by which sitagliptin treatment ameliorates dyslipidemia-related renal injury.

## 4. Experimental Section

### 4.1. Animals

All animal experiments were performed in accordance with the Institutional Animal Care Guidelines (NFYY-2013-26, 26 February 2013, Nanfang Hospital Animal Ethic Committee, Guangzhou, China). Six-week-old male C57BL/6J mice and apoE^−/−^ mice were obtained from Southern Medical University (Guangdong, China) or the Joslin Diabetes Center (Boston, MA, USA), respectively. Mice were housed in an animal care facility and were maintained on a 12-h light/dark cycle in a room controlled for temperature and humidity. Additionally, mice were allowed free access to standard chow and water. After 2 weeks of access to standard chow, the mice were divided into three groups. ApoE^−/−^ mice were randomly divided into an apoE^−/−^ group (*n* = 10) and were fed a HFD (21.8% fat, 1.25% cholesterol, 76.95% standard chow) or a sita + apoE^−/−^ group (*n* = 7) and were fed HFD mixed with 0.3% sitagliptin (200 mg/kg/d; Merck, Madison, NJ, USA). The control group consisted of 8-week-old male C57BL/6J mice that were fed a HFD (*n* = 8). All mice were fed this diet for an additional 16 weeks. At the end of experiment, mice were anesthetized by an intraperitoneal injection of 0.8% pentobarbital sodium (40 mg/kg body weight).

### 4.2. Analysis of Body Weight and Metabolic Profile

Starting at 8 weeks of age, the body weight of each mouse was measured and recorded weekly. On week 15, a intraperitoneal glucose tolerance test (IPGTT) was performed by glucose monitoring at 0, 15, 30, 60 and 120 min after intraperitoneal injection of glucose, using a One Touch SureStep glucometer (Johnson & Johnson, New Brunswick, NJ, USA). On week 16, an intraperitoneal insulin tolerance test (IPITT) was performed also by measuring blood glucose at 0, 15, 30 and 60 min after intraperitoneal injection of insulin (Johnson & Johnson). Blood samples were collected by eyeball extirpating and separated serums were stored at −80 °C until they were used in future experiments. HDL, LDL, VLDL, TG and CHOL were measured using an automatic biochemical analyzer (Dimension, Wilmington, DE, USA).

### 4.3. Assessment of Albuminuria and Urinary 8-OHdG

At 16 weeks of age, the effects of renal oxidative stress and functional injury were assessed, Mice were housed in metabolic cages with access to water and food, and urine was collected over a period of 24 h. Urinary 8-OHdG and albumin concentrations were measured using mouse enzyme-linked immunosorbent assay (ELISA) kits with anti-8-OHdG (ab101245; Abcam, Cambridge, MA, USA) or anti-albumin (ab108792; Abcam) antibodies. ELISAs were performed according to the manufacturer’s protocol.

### 4.4. Renal Pathological Changes

Kidney samples were collected and either fixed in 4% paraformaldehyde or snap frozen in liquid nitrogen. Samples were embedded in paraffin or OCT and were cut into slices using a microtome (Leica RM 2235 or Leica CM1850UV; Leica, Solms, Germany). Slices were then mounted onto glass slides and histological examinations were performed. To examine the accumulation of glomerular mesangial matrix, tissue paraffin sections were stained with PAS (Loogene, Beijing, China) and glomerular analysis was performed. Frozen sections were used to evaluate lipid deposition using Oil-red O staining (Sigma, Santa Clara, CA, USA). Tissue paraffin sections were stained with Sirius red (Loogene) and area of collagen fiber proliferation in the renal interstitium was assessed. All sections were observed under an Olympus B ×40 upright light microscope (Olympus, Tokyo, Japan). For each mouse, five fields of view were obtained in a blinded manner and evaluated using Image Pro-plus 6.0 software (Media Cybernetics, Bethesda, MD, USA).

### 4.5. cDNA Synthesis and Real-Time PCR

Total RNA was extracted from renal cortex tissues of mice using an E.Z.N.A Total RNA Kit (Omega, Norcross, GA, USA), according to the manufacturer’s instruction. The quality and concentration of extracted RNA were assessed for each sample by determining the OD 260/280 nm absorbance ratio. Only samples with a 260/280 ratio between 1.8 and 2.1 were used in subsequent experiments. Reverse transcription was performed on total RNA using the PrimerScript TM RT reagent Kit (Takara Biotechnology, Shiga, Japan). Quantitative real-time PCR was performed on generated cDNA using an ABI 7500 Real-Time PCR System (Life Technologies, Carlsbad, CA, USA). Primer sequences were as follows: *DPP-4* forward: 5'-GTCTAAGCGAGGGGAGAGAAAC-3', reverse: 5'-CAAGGCGGAGAAAGAAAGTG-3'; *GLP-1R* forward: 5'-TGACCGACTGTTTGTTCTCTTG-3', reverse: 5'-CCAACTTATGACCTTCTGGTGAC-3'; *TGF-β1* forward: 5'-GTCACTGGAGTTGTACGGCA-3', reverse: 5'-TCATGTCATGGATGGTGCCC-3'; *FN* forward: 5'-ATGAGAAGCCTGGATCCCCT-3', reverse: 5'-GGAAGGGTAACCAGTTGGGG-3'; *GAPDH* forward: 5'-GTGAAGCAGGCATCTGAGGG-3', reverse: 5'-CGAAGGTGGAAGAGTGGGAGT-3' (Invitrogen, Norcross, GA, USA). The PCR reaction contained 2 µL of template cDNA, 0.8 µM/µL of the appropriate forward and reverse primer, 10 µL of SYBR Premix Ex Taq (Takara), 0.4 µL of ROX Reference Dye II (Takara Biotechnology) and 6 µL of nuclease-free water. Real-time PCR conditions were as follows: 30 s at 95 °C followed by 40 cycles of 5 s at 95 °C and 60 °C for 34 s, then 95 °C for 15 s, 60 °C for 1 min, 95 °C for 15 s. The relative expression of each gene was calculated by the comparative 2^−∆∆*C*t^ method, using GAPDH as a reference gene. Expression values for each gene were reported as fold change over values obtained for the control group.

### 4.6. Western Blotting for Kidney Tissue

Proteins were extracted from renal cortical tissues using radioimmunoprecipitation assay buffer (P0013B; Beyotime, Shanghai, China). Samples were electrophoresed on 10% SDS-PAGE gel and proteins were transferred to polyvinylidene fluoride membrane (Immobilon, Millipore, Billerica, MA, USA). Membranes were blocked in Tris-buffered saline with 0.1% Tween-20 (TBST) containing 5% skim milk, and then were incubated in primary antibody diluent (P0023A; Beyotime) and gently shaken overnight at 4 °C. Primary antibodies against phospho-AMPK (1:1000; 2535; Cell Signaling Technologies, Danvers, MA, USA) and total AMPK (1:1000; 2532; Cell Signaling Technologies), phospho-Akt (1:1500; BS4006; Bioworld, Louis Park, MN, USA) and total Akt (1:1500; BS1008; Bioworld), TNF-β1 (1:1000; 7794-1; Epitomics, Burlingame, CA, USA), FN (1:1000; 1574-1; Epitomics), phospho-p38 (1:1000; BS4766; Bioworld) and total p38 (1:1000; BS3576; Bioworld), phospho-ERK (1:1000; 4348; Cell Signaling Technologies) and total ERK (1:1000; BS1112; Bioworld) were used. Blots were washed in TBST and then incubated with a fluorescent secondary antibody at room temperature for 1 h (1:15,000; 926-32211; LI-COR Biosciences, Lincoln, NE, USA). An antibody to β-actin was used as a loading control (1:1000; AP0060; Bioworld). Fluorescent micrographs were obtained using an Odyssey Western Blotting Kit (LI-COR Biosciences) and densitometry was performed using Gel-pro software (Media Cybernetics).

### 4.7. Statistical Analysis

All data were presented as mean ± standard deviation (S.D.). Statistical analysis was performed using SPSS software version 13.0 (SPSS Inc., Chicago, IL, USA) and Graph Pad prism software version 5.0 (GraphPad Software, La Jolla, CA, USA). Inter-group variation was measured by one-way analysis of variance testing, followed by a least-significant difference post hot test. The minimal level for statistical significance was *p* < 0.05.

## 5. Conclusions

In conclusion, our study exhibited an explicit relationship between dyslipidemia and renal injury. This is the first study to demonstrate that sitagliptin treatment is effective in attenuating dyslipidemia-related renal injury. This protective effect may be associated with the activation of AMPK and the inhibition of Akt, TGF-βl, and FN, as well as the p38/ERK MAPK signaling pathway. Additionally, this protective effect of sitagliptin appears to be distinct from its beneficial effects on glucose and lipid metabolism, and may account for enhanced GLP-1 levels. A detailed mechanistic understanding of how sitagliptin reduces the effects of dyslipidemia remains unclear and will be addressed in future studies.
